# Supplementary material for: Multi‐dataset identification of innovative feature genes and molecular mechanisms in keratoconus
Source: J Cell Mol Med. 2024 Sep 19;28(18):e70079. doi: 10.1111/jcmm.70079 (PMC11412914; doi:10.1111/jcmm.70079)
Supplement: Supplementary file 2 — Material S2. [file JCMM-28-e70079-s002.zip › 7.GSEA analysis/4.MYRF/KEGG_GLYCEROLIPID_METABOLISM.html]

Details for gene set KEGG\_GLYCEROLIPID\_METABOLISM[GSEA]

|  || Dataset | MYRF.group.cls#h\_versus\_l |
| Phenotype | group.cls#h\_versus\_l |
| Upregulated in class | h |
| GeneSet | KEGG\_GLYCEROLIPID\_METABOLISM |
| Enrichment Score (ES) | 0.2340355 |
| Normalized Enrichment Score (NES) | 0.8155364 |
| Nominal p-value | 0.79014987 |
| FDR q-value | 1.0 |
| FWER p-Value | 1.0 |
Table: GSEA Results Summary

  

Fig 1: Enrichment plot: KEGG\_GLYCEROLIPID\_METABOLISM      
 Profile of the Running ES Score & Positions of GeneSet Members on the Rank Ordered List

  

| SYMBOL | TITLE | RANK IN GENE LIST | RANK METRIC SCORE | RUNNING ES | CORE ENRICHMENT || 1 | GPAM | na | 984 | 0.463 | 0.0405 | Yes |
| 2 | GK2 | na | 2446 | 0.326 | 0.0469 | Yes |
| 3 | DGKB | na | 3125 | 0.289 | 0.0703 | Yes |
| 4 | GLYCTK | na | 3410 | 0.276 | 0.1031 | Yes |
| 5 | DGKH | na | 3859 | 0.259 | 0.1287 | Yes |
| 6 | CEL | na | 4729 | 0.230 | 0.1379 | Yes |
| 7 | PNPLA3 | na | 4763 | 0.230 | 0.1711 | Yes |
| 8 | AGPAT4 | na | 5017 | 0.222 | 0.1968 | Yes |
| 9 | DGKZ | na | 5852 | 0.197 | 0.2020 | Yes |
| 10 | PNLIPRP2 | na | 6859 | 0.168 | 0.1980 | Yes |
| 11 | DGAT2 | na | 7078 | 0.162 | 0.2158 | Yes |
| 12 | DGKD | na | 8089 | 0.136 | 0.2069 | Yes |
| 13 | DGKE | na | 8154 | 0.135 | 0.2251 | Yes |
| 14 | MBOAT2 | na | 8729 | 0.122 | 0.2266 | Yes |
| 15 | MBOAT1 | na | 9065 | 0.115 | 0.2340 | Yes |
| 16 | AGPAT3 | na | 10594 | 0.085 | 0.2026 | No |
| 17 | AKR1A1 | na | 11160 | 0.076 | 0.1975 | No |
| 18 | ALDH3A2 | na | 11227 | 0.075 | 0.2067 | No |
| 19 | DGKQ | na | 11500 | 0.070 | 0.2094 | No |
| 20 | AGPAT2 | na | 12005 | 0.063 | 0.2042 | No |
| 21 | LCLAT1 | na | 13043 | 0.050 | 0.1817 | No |
| 22 | AGK | na | 13599 | 0.043 | 0.1721 | No |
| 23 | LPL | na | 16101 | 0.017 | 0.1025 | No |
| 24 | DGAT1 | na | 16225 | 0.015 | 0.1012 | No |
| 25 | AGPAT1 | na | 17745 | 0.002 | 0.0577 | No |
| 26 | ALDH7A1 | na | 19743 | -0.019 | 0.0029 | No |
| 27 | PNLIPRP1 | na | 20729 | -0.030 | -0.0210 | No |
| 28 | DGKA | na | 21166 | -0.035 | -0.0283 | No |
| 29 | LIPG | na | 21448 | -0.039 | -0.0306 | No |
| 30 | PNLIP | na | 21992 | -0.046 | -0.0395 | No |
| 31 | LIPF | na | 23085 | -0.061 | -0.0619 | No |
| 32 | ALDH9A1 | na | 23662 | -0.070 | -0.0681 | No |
| 33 | GPAT2 | na | 24195 | -0.079 | -0.0717 | No |
| 34 | GLA | na | 24509 | -0.084 | -0.0682 | No |
| 35 | ALDH1B1 | na | 25124 | -0.095 | -0.0717 | No |
| 36 | LIPC | na | 26838 | -0.131 | -0.1016 | No |
| 37 | DGKG | na | 26891 | -0.133 | -0.0834 | No |
| 38 | MGLL | na | 30031 | -0.224 | -0.1405 | No |
| 39 | GK | na | 30912 | -0.259 | -0.1274 | No |
| 40 | DGKI | na | 31832 | -0.309 | -0.1080 | No |
| 41 | AKR1B1 | na | 32075 | -0.321 | -0.0672 | No |
| 42 | AWAT2 | na | 32555 | -0.354 | -0.0283 | No |
| 43 | ALDH2 | na | 34342 | -0.612 | 0.0111 | No |
Table: GSEA details [plain text format]

  

Fig 2: KEGG\_GLYCEROLIPID\_METABOLISM      
 Blue-Pink O' Gram in the Space of the Analyzed GeneSet

  

Fig 3: KEGG\_GLYCEROLIPID\_METABOLISM: Random ES distribution      
 Gene set null distribution of ES for **KEGG\_GLYCEROLIPID\_METABOLISM**
